# Intestinal Microbial Metabolites Are Linked to Severity of Myocardial Infarction in Rats

**DOI:** 10.1371/journal.pone.0160840

**Published:** 2016-08-09

**Authors:** Vy Lam, Jidong Su, Anna Hsu, Garrett J. Gross, Nita H. Salzman, John E. Baker

**Affiliations:** 1 Division of Cardiothoracic Surgery, Medical College of Wisconsin, Milwaukee, WI, United States of America; 2 Department of Pharmacology and Toxicology, Medical College of Wisconsin, Milwaukee, WI, United States of America; 3 Division of Pediatric Gastroenterology, Medical College of Wisconsin, Milwaukee, WI, United States of America; 4 Department of Biochemistry, Medical College of Wisconsin, Milwaukee, WI, United States of America; 5 The Cardiovascular Center, Medical College of Wisconsin, Milwaukee, WI, United States of America; Emory University, UNITED STATES

## Abstract

Intestinal microbiota determine severity of myocardial infarction in rats. We determined whether low molecular weight metabolites derived from intestinal microbiota and transported to the systemic circulation are linked to severity of myocardial infarction. Plasma from rats treated for seven days with the non-absorbed antibiotic vancomycin or a mixture of streptomycin, neomycin, polymyxin B and bacitracin was analyzed using mass spectrometry-based metabolite profiling platforms. Antibiotic-induced changes in the abundance of individual groups of intestinal microbiota dramatically altered the host’s metabolism. Hierarchical clustering of dissimilarities separated the levels of 284 identified metabolites from treated vs. untreated rats; 193 were altered by the antibiotic treatments with a tendency towards decreased metabolite levels. Catabolism of the aromatic amino acids phenylalanine, tryptophan and tyrosine was the most affected pathway comprising 33 affected metabolites. Both antibiotic treatments decreased the severity of an induced myocardial infarction *in vivo* by 27% and 29%, respectively. We then determined whether microbial metabolites of the amino acids phenylalanine, tryptophan and tyrosine were linked to decreased severity of myocardial infarction. Vancomycin-treated rats were administered amino acid metabolites prior to ischemia/reperfusion studies. Oral or intravenous pretreatment of rats with these amino acid metabolites abolished the decrease in infarct size conferred by vancomycin. Inhibition of JAK-2 (AG-490, 10 μM), Src kinase (PP1, 20 μM), Akt/PI_3_ kinase (Wortmannin, 100 nM), p44/42 MAPK (PD98059, 10 μM), p38 MAPK (SB203580, 10 μM), or K_ATP_ channels (glibenclamide, 3 μM) abolished cardioprotection by vancomycin, indicating microbial metabolites are interacting with cell surface receptors to transduce their signals through Src kinase, cell survival pathways and K_ATP_ channels. These inhibitors have no effect on myocardial infarct size in untreated rats. This study links gut microbiota metabolites to severity of myocardial infarction and may provide future opportunities for novel diagnostic tests and interventions for the prevention of cardiovascular disease.

## Introduction

Ischemic heart disease is the leading cause of morbidity and mortality in all industrialized nations. An estimated 1 million Americans will have a new or recurrent acute myocardial infarction each year [1], with many survivors experiencing lasting morbidity, progression to heart failure and death. Because of the many strong mechanistic links between a diet rich in lipids and the progression to cardiovascular disease and acute myocardial infarction, therapeutic advances have focused primarily on reduction in either ingestion or synthesis of cholesterol, and reduction in dietary trans and saturated fatty acids and triglycerides. Despite this, even in the setting of aggressive high potency statin therapy and global cardiovascular risk reduction efforts, most clinical trials reveal a significant residual cardiovascular risk with, at best, only a 30% reduction in major adverse cardiovascular events. Therefore, there exists a significant unmet clinical need for identifying novel therapies for the prevention and treatment of acute myocardial infarction. Development of such potential therapies requires identification of additional contributory processes that determine severity of myocardial infarction in order that mechanism based interventions may be developed.

Humans and other animals are colonized by complex ecosystems of microbes. The vast majority of these microbes (tens of trillions), collectively termed the microbiome, live in our gastrointestinal tract. There are 500–1000 bacterial species living in the human intestines, and the gene content of microbes in the human gut exceeds that of the host by 100-fold [2]. The intestinal microbiota is essential for human and animal health. Disruption of the intestinal microbiota can promote the development of complex metabolic diseases such as obesity [3] and atherosclerosis [4].

A direct link between the intestinal microbiota and the severity of injury from an induced myocardial infarction in rats has been reported [5]. In this study, the broad-spectrum antibiotic vancomycin altered the abundance of individual groups of intestinal microbiota and decreased circulating leptin levels, resulting in smaller myocardial infarcts and improved recovery of post ischemic mechanical function. In addition, the leptin suppressing probiotic bacterium, *Lactobacillus plantarum 299v* (live microorganism beneficial to its host), that lives in the intestines, also resulted in decreased leptin levels, smaller myocardial infarcts and greater recovery of post-ischemic mechanical function. These antibiotic and probiotic treatments appear to cause a shift in the intestinal microbial population towards a cardioprotective phenotype. This study demonstrates that antibiotic and probiotic treatments could extend positive influences of the intestinal microbiota far beyond merely local effects to create positive, significant impacts on remote organs such as the heart.

In addition to leptin, low-molecular weight metabolites produced and metabolized by intestinal microbiota are continuously being absorbed from the intestinal lumen. Following absorption, the metabolites are transported to the liver for processing and then released into the systemic circulation. Once there, they can provide benefit and/or cause harm to the host, for example, in the promotion or reduction of cardiovascular disease [4]. A mechanistic link between metabolites that are regulated by the intestinal microbiota and the severity of myocardial infarction has not yet been reported. Discovery of a relationship between intestinal microbiota, microbial metabolites present in the circulation, and severity of myocardial infarction would mean that for the first time, we may be able to detect a person’s likelihood of having a heart attack by addressing non-conventional risk factors. The link between intestinal microbiota and the severity of heart attacks may also provide opportunities for novel therapeutic approaches to prevent heart attacks.

Mass spectrometry has become a powerful tool for metabolomic studies due to its wide dynamic range, reproducible quantitative capabilities and its ability to analyze samples of significant molecular complexity [6]. Data analysis involves the mining of high-density spectral data generated from biofluids such as blood plasma measured against a set of reference biochemical signatures using computer pattern recognition algorithms. Mass spectrometry has been used to compare and contrast the differences in blood plasma metabolites of conventional and germ-free mice [7], and to characterize gastrointestinal bacterial function [8]. These studies utilized a broad, untargeted mass spectrometry approach to reveal the identity of multiple metabolites. However, these studies did not link microbiota-induced metabolic changes with physiologic function in the host.

We have utilized mass spectrometry for a hypothesis testing study to determine whether intestinal microbiota metabolic phenotypes are linked to severity of myocardial infarction, and to comprehensively determine the presence of metabolic phenotypes in the circulation of the host induced by non-absorbed antibiotics. We chose to analyze plasma to assess the extent of interplay between microbial metabolic and systemic pathways.

The identity of the sites targeted by microbial metabolites in the heart and the corresponding intracellular pathways that decrease severity of myocardial infarction are not known. Activation of intracellular survival pathways may be initiated by receptor binding and effected by ion channels located on the surface or inside of cells [9]. An understanding of how microbial metabolites communicate with the heart and the identity of the cellular mediators responsible for decreasing severity of myocardial infarction is needed to determine the underlying cardioprotective mechanism.

We tested the hypothesis that low molecular weight metabolites produced by intestinal microbiota and carried to the systemic circulation play a direct role in determining severity of myocardial infarction. The major objectives were to (i) identify plasma metabolites and how they respond to antibiotic treatments, (ii) determine subsequent severity of injury from myocardial ischemia/reperfusion, (iii) evaluate whether gut microbiota metabolites play a physiological role in determining severity of myocardial infarction and, (iv) determine the cellular mediators of cardioprotection. Our study mechanistically links gut microbiome metabolic phenotypes to the severity of myocardial infarction in rats.

## Methods

**Fig 1:**
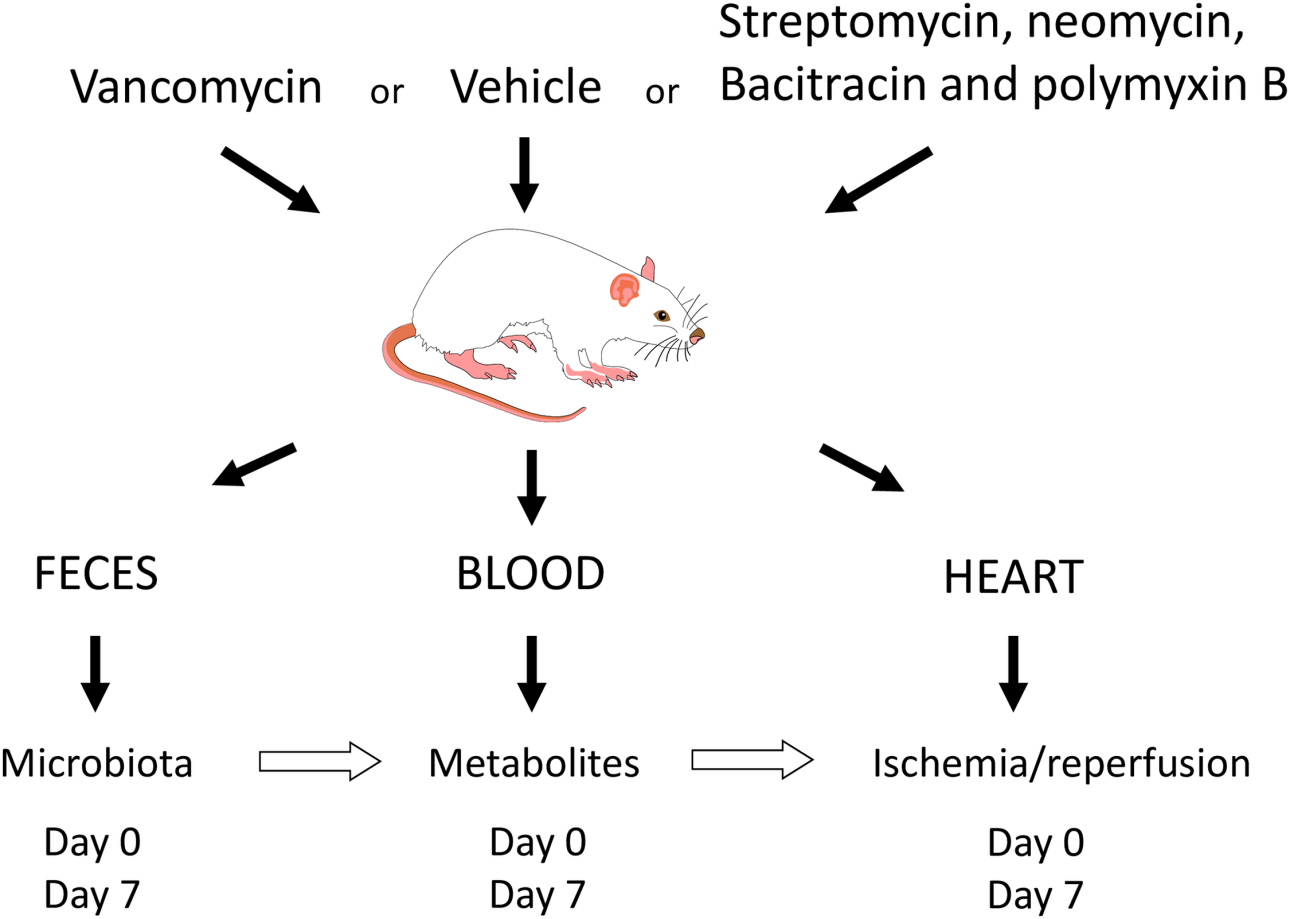
Protocol to examine the relationship between the intestinal microbial metabolites and severity of myocardial infarction.

The experimental design for the study is shown in Fig 1.

### Rat handling and antibiotic treatment

Rats received humane care in compliance with the U.S National Institutes of Health Guide for the Care and Use of Laboratory Animals (NIH Publication No. 85–23, revised 1996). Rat handling and use protocols were approved by the Institutional Animal Care and Use Committee at the Medical College of Wisconsin (AUA 3499). Group sizes were equal. The numbers in each experimental group were based on our previous experience with the same measurements in similar studies. Animals were randomized to each group. The analysis was carried out in a blinded manner. Male Dahl S rats (200–220 g; Charles River, Wilmington, MA, USA) were fed autoclavable laboratory rodent diet 5010 (LabDiet, St. Louis, MO, USA) and given water *ad libitum* before antibiotic treatment. Vancomycin (60 mg/kg/day) or a combination of streptomycin (120 mg/kg/day), neomycin (60 mg/kg/day), bacitracin (120 mg/kg/day), and polymyxin B (60 mg/kg/day), antibiotics known to alter gastrointestinal microbiota in mice [10], were added to the drinking water. Rats were anesthetized by intraperitoneal injection of pentobarbital (Nembutal; 50 mg/kg). While anesthetized, the rats were monitored for anesthetic depth *via* assessments of the pedal reflex and respiratory rate. Blood was collected from the jugular vein in EDTA coated tubes kept on ice. Plasma was obtained by centrifuging the blood tubes at 1000 g within 30 minutes of collection. At the end of the experiments, rats were euthanized with an overdose of intraperitoneal pentobarbital and a pneumothorax performed.

### Metabolomic studies

Blood was withdrawn on days 0 and 7 for metabolite analysis. The metabolites present in the blood of control and antibiotic-treated rats were characterized using gas chromatography/mass spectrometry and liquid chromatography-tandem mass spectrometry platforms (Metabolon, Durham, NC). Compounds were identified by comparison to library entries of purified standards or recurrent unknown entities as described in Supporting Information (S1 File). A detailed description of the mass spectrometry analysis is provided in Supporting Information (S1 File).

### Ischemia/reperfusion studies *in vivo*

An *in vivo* anesthetized rat model was used for these experiments using the general surgical protocol and determination of infarct size (IS) described previously [11]. Briefly following anesthesia, a tracheotomy for artificial ventilation was performed, with the left common carotid artery cannulated for blood pressure and heart rate measurements. A thoracotomy was performed at the fifth intercostal space, the pericardium excised, and a silk ligature placed distal to the left atrial appendage spanning the sternal portion of the left ventricle including the left anterior descending coronary artery. Occlusion of the area described [area at risk (AAR)] was created by placing the ends of the ligature through a polypropylene tube and fixing the snare to the epicardial surface with a hemostat. After 30 min, the hemostat was released to reperfuse the AAR. Following 3 h of reperfusion, the ligature was again occluded and the AAR was determined by patent-blue negative staining. The heart was then excised, cross sectioned into 4–5 thin pieces along the long axis, from apex to base, and separated into normal zone and AAR. The pieces were incubated in 1% 2,3,5-triphenyltetrazolium chloride (TTC) in 100 mM phosphate buffer (pH 7.4) and incubated at 37°C for 15 min. The heart was then incubated overnight in 10% formaldehyde, and the infarcted tissue was dissected from the AAR under the illumination of a dissecting microscope (Cambridge Instruments). IS and AAR were determined by gravimetric analysis. IS was expressed as a percentage of the AAR (IS/AAR).

### Ischemia/reperfusion studies *in vitro*

Hearts were perfused in the Langendorff mode at a constant perfusion pressure of 90 mmHg as described previously [12]. Briefly, hearts were perfused with modified Krebs-Henseleit buffer (120 mM NaCl, 25 mM NaHCO_3_, 4.7 mM KCl, 1.2 mM KH_2_PO_4_, 1.20 mM MgSO_4_, 11 mM glucose, and 1.8 mM CaCl_2_) bubbled with 95% O_2_-5% CO_2_ for a 40-min stabilization period and subjected to 25 min of global no-flow ischemia, followed by 180 min of reperfusion. Before use, all perfusion fluids were filtered through cellulose acetate membranes with pore size of 5.0 μm to remove particulate matter.

The hearts were kept in temperature-controlled chambers to maintain myocardial temperature at 37°C. A balloon connected to a pressure transducer was inserted into the left ventricle to monitor cardiac function. Left ventricular end diastolic pressure (LVEDP) was set to 5–6 mmHg. For some experiments, hearts were stabilized for 25 min and then perfused with vancomycin for 15 min before ischemia/reperfusion. During the initial 40-min reperfusion period, recovery of mechanical function was measured as left ventricular developed pressure (LVDP) under steady-state conditions and expressed as a percentage of pre-ischemic LVDP. The increase in LVEDP during reperfusion was measured and expressed in mmHg. Following 3 hours of reperfusion, 1.0%(w/v) TTC was delivered to the isolated heart *via* a syringe attached to the 3-way stopcock for IS determination. TTC was administered at a rate of 1 ml/min for 10 min. The left ventricle was then isolated and sliced along the long axis, from apex to base, into 4–5 thin cross-sectional pieces. The tissues were then incubated for 15 min in a 1% TTC solution in 100 mM phosphate buffer (pH 7.4) at 37°C. Tissues were then stored in 10% formaldehyde overnight and the extent of infarcted myocardium was measured. The infarcted tissue was then separated from the non-infarcted tissue and measured as a percentage (% Infarct = Infarct Area/Area at Risk) × 100 using a fluorescent, digital, color camera with a 55 mm lens (Nikon). Imaging analysis software (Imaging Research, St. Catharines, ON) was used to recognize infarcted tissue (scan area) in proportion to entire LV (total target area).

### Amino acid metabolite treatment

Untreated and vancomycin-treated rats were administered metabolites of phenylalanine [trans-cinnamate (4.50 μg/kg), phenylacetate (4.08 μg/kg), and 3-phenylpropionate (3.06 μg/kg) acids], tryptophan [indole-3-acetic acid (0.26 μg/kg), 3-indoxyl sulfate (124.50 μg/kg), L-kynurenine (34.99 μg/kg) and 3-indolepropionic acid (2.73 μg/kg)] and tyrosine [4-hydroxyphenylpruvic acid (2.04 μg/kg), and p-hydroxyphenyllactic acid (3.54 μg/kg)] intravenously at 24 and 12 hours prior to ischemia/reperfusion studies *in vitro*. For oral treatments, the same amount of metabolites were dissolved in the drinking water and given *ad libitum* (each rat drank an average of 15 ml/day) 48 hours prior to the ischemia/reperfusion studies.

### Fecal microbiota abundance

Fresh fecal pellets were obtained from each rat before (day 0) and at day 7 post treatment. Pellets were homogenized in 1 ml PBS, and 200 μl of the homogenate was used for microbial DNA isolation using the QIAamp DNA Stool Mini Kit (Qiagen, Valencia, CA, USA). Isolated DNA samples were subjected to quantitative PCR using an iCycler (Bio-Rad, Hercules, CA, USA) for microbial population enumeration. The PCR reaction mixture consisted of 50% iQ SYBR Green Supermix (Bio-Rad), 0.4 μM forward and reverse primers, and 3.8% template solution in RNase/DNase-free water. Primer sets specific for the 16S and 18S rRNA of particular microbial phylum, class, genus and species (*Methanobrevibacter smithii* and *L*. *plantarum*) along with reaction temperature and reference strains are detailed in Supporting Information (S3 Table) [10, 13–22].

### Statistical analysis

Bacterial densities were log_10_ transformed, and a paired, 2-tailed, *t* test used to determine the significance of any differences. Data reported were means ± SD from 6–8 animals in each group. Significance was set at *P* < 0.05. For the metabolite levels, mean and median values were reported. For pair-wise comparisons in the metabolite levels, Welch’s t-tests and/or Wilcoxon’s rank sum tests were used. Significance was assigned for differences with p-value ≤ 0.05. False discovery rates were estimated using Storey J. D. and Tibshirani R.’s method [23]. For all 193 tests with p-values of ≤ 0.05, q-values were reasonably low at ≤0.03 suggesting less than 6 were false positives. We thus set q-value cut off at 0.03.

## Results

### Antibiotics and abundance of individual groups of gut microbiota

**Fig 2:**
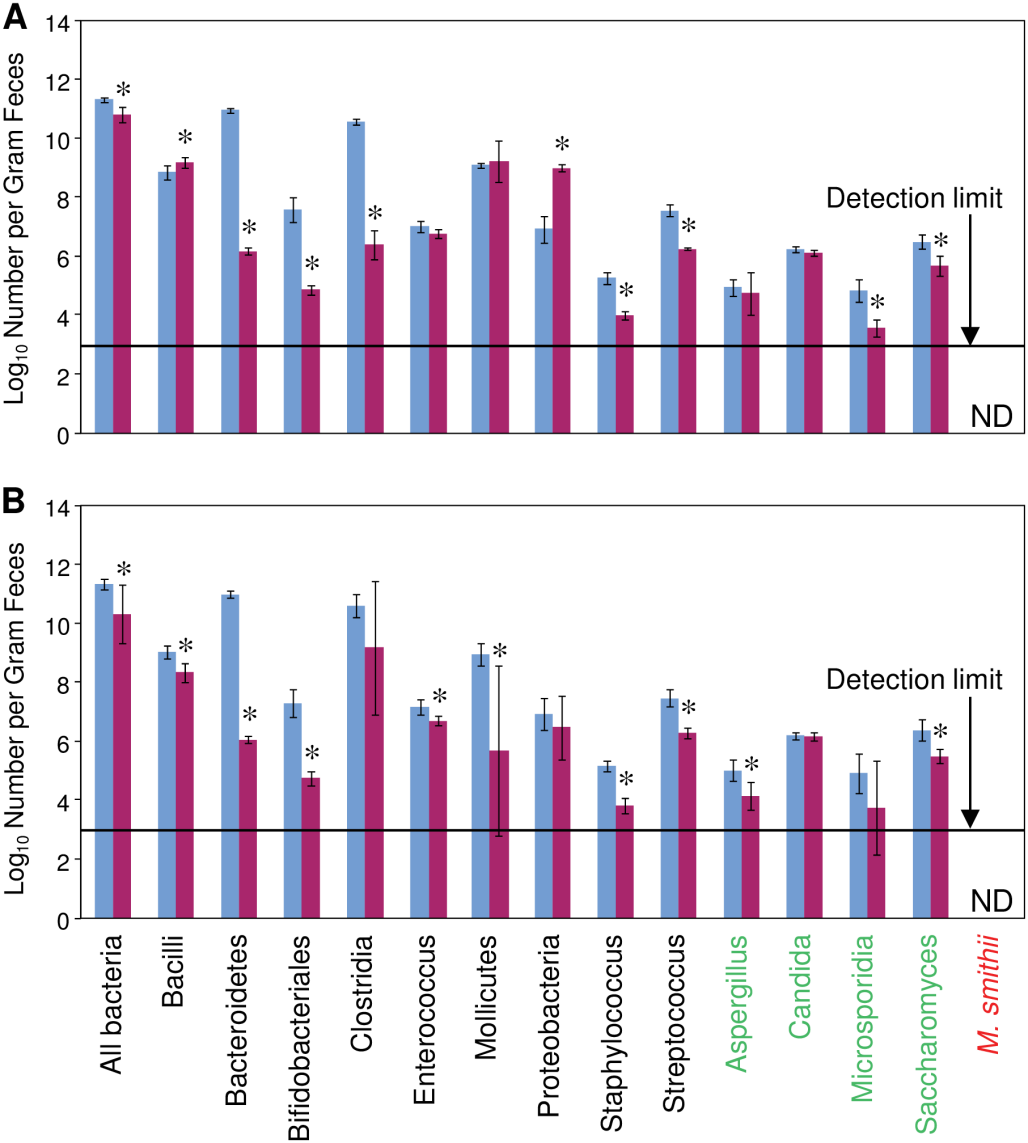
Microbial populations in the feces of antibiotic treated rats. (A) Vancomycin (60mg/kg/day) and (B) a mixture of streptomycin (120 mg/kg/day), neomycin (60 mg/kg/day), polymyxin B (60 mg/kg/day), and bacitracin (120 mg/kg/day) administered orally reduced total microbial numbers and altered abundance of microbial taxa. Microbial abundance was determined using PCR. The X-axis labels show taxa of microbes grouped by bacteria (black), fungi (green), and archaea (red). Data are mean ±SD, n = 6/group. * p ≤ 0.05; day 0 vs. day 7. ND = not detected. The results from the vancomycin treatment in Fig 2A are reproduced with permission from reference 5.

To alter the composition of the intestinal microbiota, a mixture of minimally absorbed antibiotics (streptomycin, neomycin, bacitracin and polymyxin B) or a single antibiotic, vancomycin, was added to the drinking water for 7 days. The rats remained healthy with no loss in body weight during the antibiotic treatments. The microbial populations present in the feces were monitored by 16S/18S rRNA quantitative RT-PCR. The primers used uniquely targeted the 16S rRNA gene of each eubacteria and archaea taxon, and the unique 18S rRNA gene of each fungal taxon. Vancomycin alone or the combination of antibiotics reduced total bacterial numbers and altered the abundance of specific bacterial and fungal groups (Fig 2). *Methanobrevibacter smithi*, a member of the archaea, was not detected in the feces. The results from the vancomycin treatment in Fig 2A are reproduced with permission from reference 5 and are included for comparative purposes with the results from the antibiotic combination treatment shown in Fig 2B.

### Mass spectrometry reveals antibiotics have a broad effect on gut microbiota metabolic phenotypes

**Fig 3:**
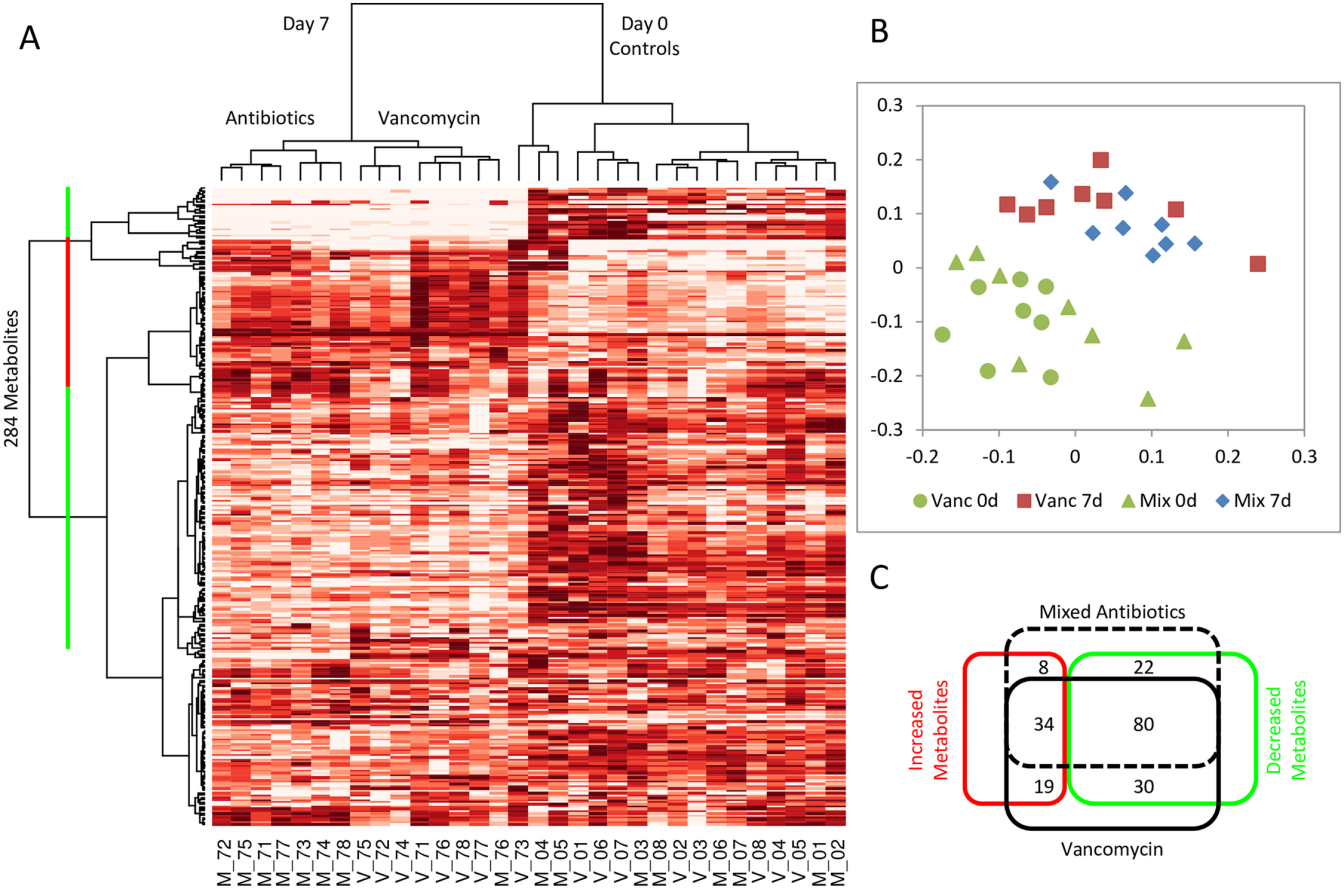
Antibiotic treatments induced characteristic changes in blood metabolites. (A) Heat map of 284 metabolites in rat plasma samples clustered by concentration profile. Sample identification: V—vancomycin treatment; M—mixed antibiotics treatment; (B) Nonmetric multidimensional scaling ordination of blood samples. (C) Venn diagram of changes in metabolite concentrations induced by the treatments.

In initial studies, we sought to discover unbiased small molecule metabolic profiles in plasma that could subsequently be used to drive hypothesis-driven identification of candidate biomarkers for severity of myocardial infarction. Both antibiotic treatments induced characteristic changes in blood metabolites. Mass spectrometry analysis identified 284 individual metabolites against a reference library of 1000 standards in the blood samples. Hierarchical clustering according to dissimilarities in the levels of the metabolites separated the 32 blood samples into three main groups: a mixed group of 16 control rats prior to drug treatment (Day 0, Fig 3A), 7 rats treated with vancomycin (Day 7 vancomycin, Fig 3A), and 7 rats treated with a mixture of streptomycin, neomycin, bacitracin, and polymyxin B (Day 7 Antibiotics, Fig 3A). The predominant phenotype of the antibiotic treatment was manifest as reduced levels of the metabolites clustered at the top and in the middle of the heat map (green bar, Fig 3A), and increased levels of the metabolites that clustered in between (red bar, Fig 3A). Two-dimensional nonmetric multidimensional scaling ordination of the samples further highlights the separation of the control and antibiotic treated groups (Fig 3B).

Of the 284 identified metabolites, 193 were significantly altered (p < 0.05) by the antibiotic treatments. The antibiotic treatments tended to reduce the levels of the metabolites; 132 of the 193 affected metabolites were reduced (p < 0.05) by vancomycin, mixed antibiotics, or both (Fig 3C). Vancomycin reduced 110 and increased 53 metabolites, while mixed antibiotics reduced 102 and increased 42 metabolites. Similar changes were observed between the two antibiotic treatments; more than half of the affected metabolites, 80 of the 132, decreased, and 34 of the 61 increased metabolites were common to both vancomycin and mixed antibiotic treatments. The comprehensive list of metabolites and their levels are shown in Supporting Information (S2 Table).

Changes in metabolites reflected changes in specific metabolic pathways. The 284 identified metabolites were mapped to eight major metabolic pathways: amino acid, peptide, carbohydrates, lipid, nucleotide, cofactors, vitamins, and xenobiotics (Supporting Information, S1 Table). Each pathway was further divided into sub-pathways (Supporting Information, S1 Table)). The general trend for each metabolic pathway was expressed as the percent of metabolites that was decreased or increased in response to either antibiotic treatment (Supporting Information, S1 Table). The majority of the metabolic pathways including amino acid, carbohydrate, energy, nucleotide, cofactors, vitamins, and xenobiotics were suppressed.

### Catabolism of aromatic amino acids is a prevalent response of gut microbiota to antibiotics

Amino acid catabolism was by far the most affected pathway. Fifty metabolites were significantly altered by one or both of the antibiotic treatments, 47 of which were significantly decreased (p < 0.05, Supporting Information, S2 Table). Metabolites of the aromatic amino acids phenylalanine, tryptophan, and tyrosine constituted the majority (33 of 50) of the affected metabolites.

**Fig 4:**
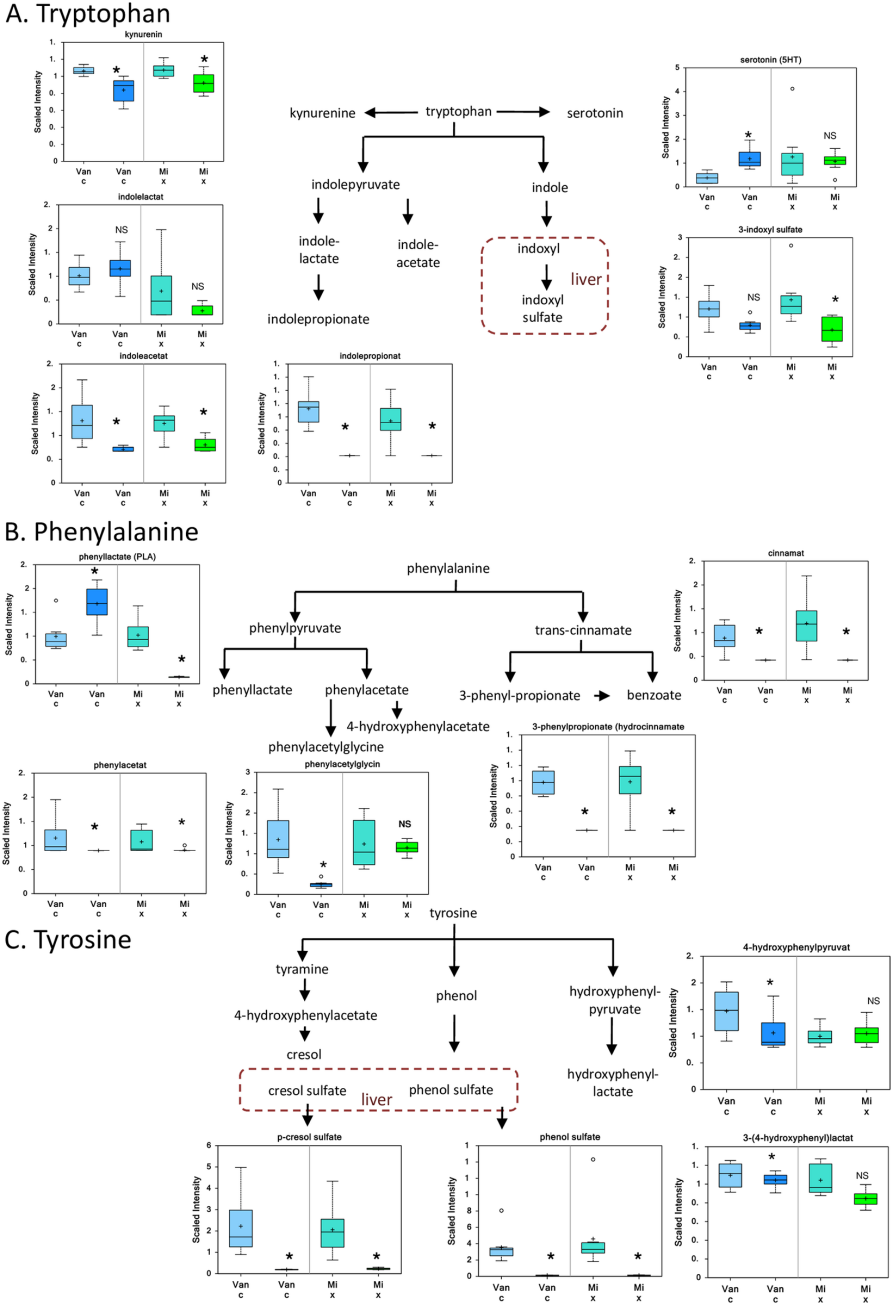
Antibiotics and intestinal microbiota metabolites. Box and whisker plots of the relative levels of metabolites derived from A. Tryptophan, B. Phenylalanine and C. Tyrosine. The mean value is represented by the plus sign. The median value is represented by the horizontal bar. The top and bottom boxes represent the upper and lower quartile. The whiskers represent the maximum and minimum values. The open circle represents an extreme data point. Data are means ±SD, n = 8/group, * = p< 0.05 vs. day 0, NS = not significant.

Treatment with vancomycin alone or a mixture of the four antibiotics decreased multiple breakdown products of the aromatic amino acids tryptophan (kynurenine, indoleacetate, indolepropionate, and 3-indoxyl sulfate) (Fig 4A); phenylalanine (phenyllactate, phenylacetylglycine, phenylacetate, 3-phenylpropionate, and cinnamate) (Fig 4B); and tyrosine (p-cresol sulfate, phenol sulfate, 3-(4-hydroxyphenyl) lactate, and 4-hydroxyphenylpyruvate) (Fig 4C). Sulfated products of the tryptophan metabolite 3-indoxyl sulfate (Fig 3A), and tyrosine metabolism p-cresol sulfate and phenol sulfate (Fig 4C), which form in the liver, were decreased following both antibiotic treatments (Fig 4C). Treatment with vancomycin alone increased serotonin and phenyllactate levels.

### Gut microbiota determine severity of myocardial infarction

**Fig 5:**
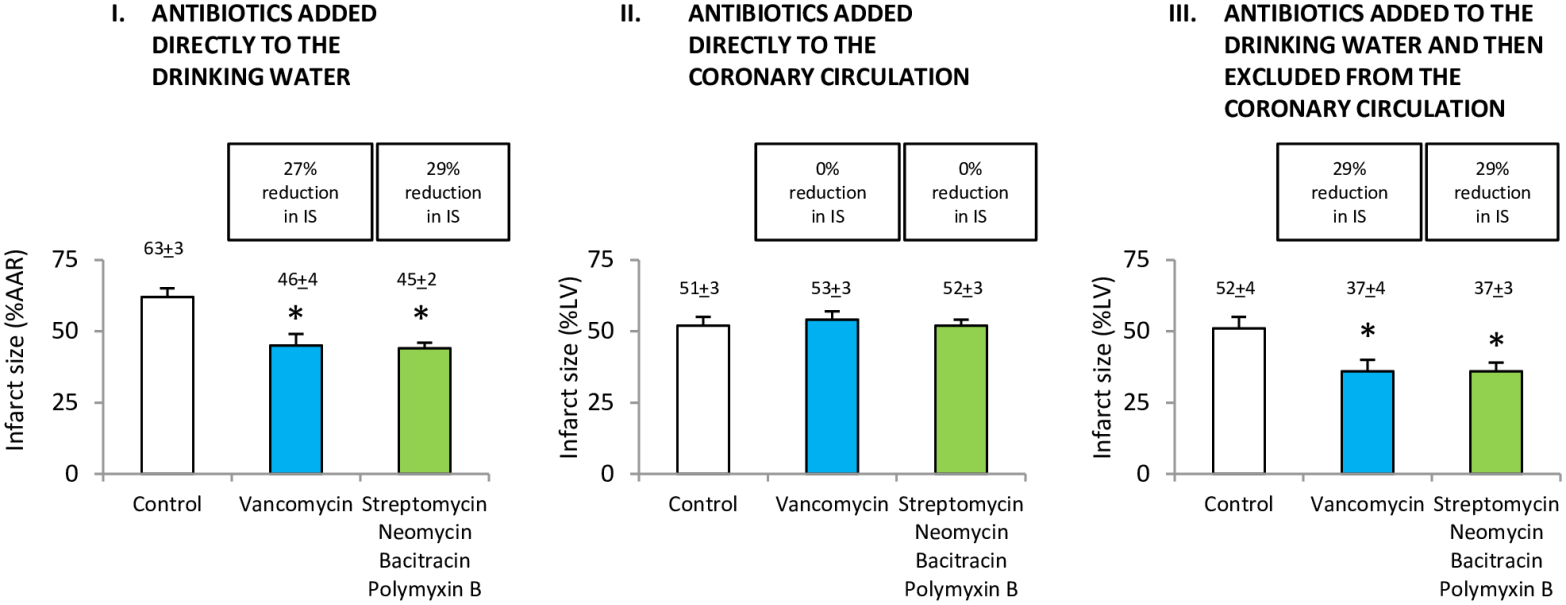
Gut microbiota determines severity of myocardial infarction. A. Antibiotics added to the drinking water reduced infarct size (IS) *in vivo*. B. Antibiotics added directly to the coronary circulation of isolated hearts did not reduce IS *in vitro*. C. Antibiotics added to the drinking water and then excluded from the coronary circulation of isolated hearts reduced IS. Data are means ± SD; *n* = 6/group. AAR, area at risk. LV, left ventricle. Reduction in infarct size was similar for *in vitro* and *in vivo* studies (A, C). *P < 0.01 *vs*. control. Representative images of rat heart slices for measurement of infarct size are shown in the Supporting Information (S1 Fig).

Vancomycin alone or the combination of antibiotics decreased myocardial infarct size *in vivo* by 27% and 29%, respectively (Fig 5A). To determine whether the decrease in infarct size was due to a direct effect of antibiotics present in the coronary vasculature, we measured blood levels of vancomycin, streptomycin, neomycin, bacitracin and polymyxin B. The level of these antibiotics in the blood was below the detection limits of the assays used (1 μM). When these antibiotics were added directly to the coronary perfusate at a concentration of 1 μM in an *in vitro* model of myocardial ischemia/reperfusion, there was no reduction in infarct size (Fig 5B). To determine further whether the decrease in infarct size was indirect, antibiotics were added to the drinking water and then excluded from the coronary perfusate in an *in vitro* model of ischemia/reperfusion. Vancomycin alone or the combination of antibiotics decreased infarct size by 29% and 29%, respectively (Fig 5C). These studies showed a reduction in myocardial infarct size despite the absence of the antibiotics in the coronary perfusate at the time of ischemia/reperfusion.

### Reconstitution of gut microbiota amino acid metabolites prevents antibiotic-induced decrease in severity of myocardial infarction

As amino acid catabolism was the most affected pathway, we determined whether the phenotype of decreased microbial metabolites of the aromatic amino acids present in the circulation was linked to decreased severity of myocardial infarction. Untreated and vancomycin-treated rats were intravenously administered metabolites of phenylalanine (trans-cinnamate, phenylacetate, and 3-phenylpropionate), tryptophan (indole-3-acetate, 3-indoxyl sulfate, L-kynurenine and 3-indolepropionate), and tyrosine (4-hydroxyphenylpruvate and p-hydroxyphenyllactate) at 24 and 12 hours prior to ischemia/reperfusion studies *in vitro*. These doses were selected to reconstitute the metabolite concentrations in the circulation.

**Fig 6:**
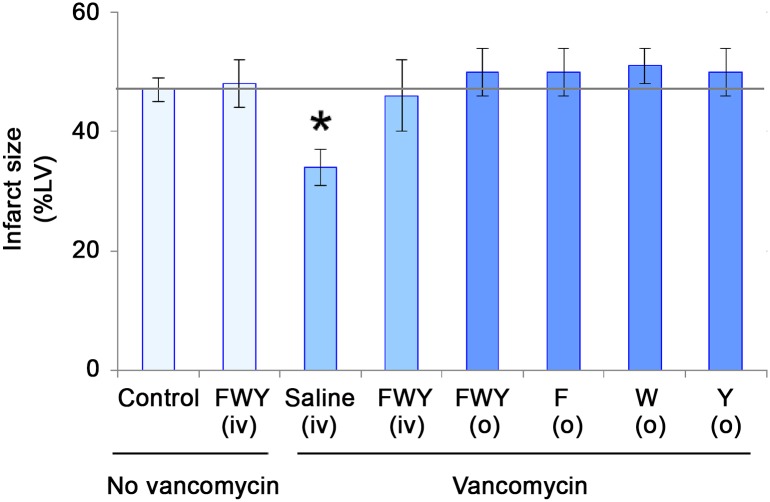
Reconstitution of gut microbiota amino acid catabolites prevents antibiotic-induced decrease in severity of myocardial infarction. Reduction of infarct size with vancomycin was abolished by pretreatment with metabolites of all three or each individual aromatic amino acid, phenylalanine (F), tryptophan (W) and tyrosine (Y). Rats treated with vancomycin were administered metabolites of phenylalanine, (Trans-cinnamate + phenylacetate + 3-phenylpropionate), tryptophan, (Indole-3-acetate + 3-indoxyl sulfate + L-kynurenine + 3-indolepropionate), or tyrosine, (4-hydroxyphenylpyruvate + p-hydroxyphenyllactate) intravenously or orally prior to ischemia/reperfusion studies. LV = left ventricle. Data are mean ± SD, n = 6/group, * = p<0.05 vs. control. iv = intravenous. o = oral.

Rats treated intravenously with metabolites of phenylalanine, tryptophan and tyrosine abolished the decrease in infarct size conferred by vancomycin treatment (Fig 6). Intravenous amino acid metabolite treatment in the absence of vancomycin administration had no effect on infarct size (Fig 6).

To further determine if dietary reconstitution of the metabolites was sufficient to abolish vancomycin-induced reduction in infarct size, the combined or individual metabolites of phenylalanine, tryptophan and tyrosine were added to the drinking water of vancomycin-treated rats for 48 hours. Oral administration of the amino acid metabolites either combined or alone abolished the reduction in infarct size conferred by vancomycin (Fig 6).

### Cellular mediators of vancomycin-induced cardioprotection

We determined whether microbial metabolites communicate with the heart *via* receptor binding and the identity of the mediators associated with decreased severity of myocardial infarction.

#### JAK-2

**Fig 7:**
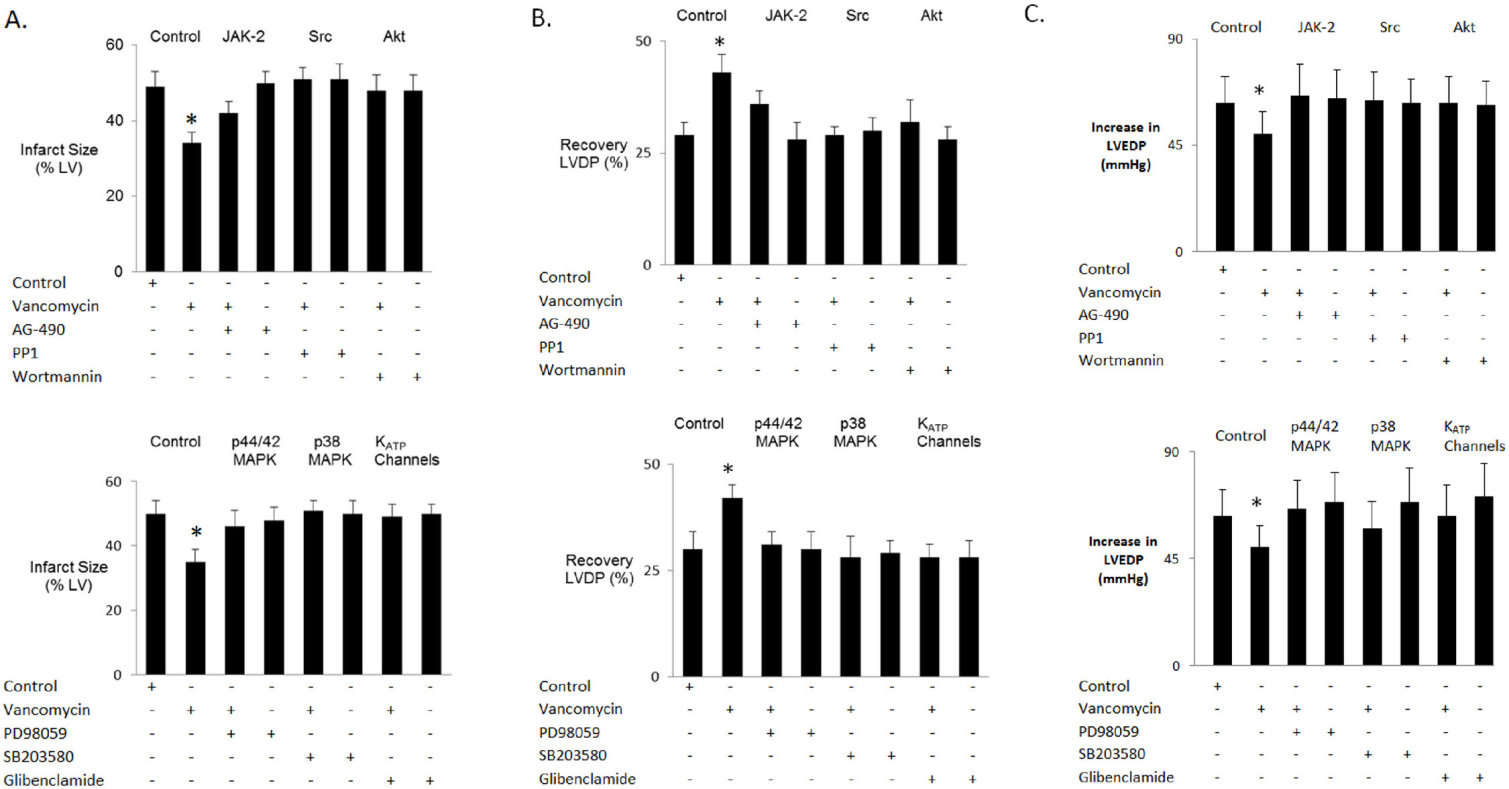
Receptors, intracellular survival pathways and ATP-dependent potassium channels activated by microbial metabolites. A: infarct size. B and C: recovery of mechanical function post reperfusion in hearts isolated from control- and vancomycin-treated rats perfused with pharmacological inhibitors of JAK-2, Src, Akt, p42/44 MAPK, p38 MAPK and K_ATP_ channels prior to ischemia/reperfusion. LV = left ventricle. LVDP = left ventricular developed pressure. LVEDP = left ventricular end diastolic pressure. Data are mean ± SD, n = 6 per group. * = p< 0.05 vs. control.

We investigated whether microbial metabolites bind to a receptor and activate Janus kinase (JAK) to play a role in cardioprotection. Hearts were isolated from vancomycin-treated rats and perfused with the JAK-2 inhibitor AG-490 (1 μM) prior to ischemia/reperfusion. AG-490 partially abolished the ability of vancomycin to reduce myocardial necrosis (Fig 7A) and to enhance ventricular function (Fig 7B) following ischemia/reperfusion. AG-490 alone had no effect on cardioprotection. AG-490 did not affect the increase in LVEDP during reperfusion (Fig 7C).

#### Src kinase

To determine whether proto-oncogene tyrosine-protein (Src) kinase plays a role in cardioprotection by vancomycin, isolated hearts were perfused with the proto-oncogene tyrosine-protein kinase inhibitor PP1 prior to ischemia and reperfusion. PP1 (20 μM) abolished the ability of vancomycin to reduce myocardial necrosis and to enhance ventricular function following ischemia/reperfusion. PP1 alone had no effect on cardioprotection (Fig 7A and 7B). PP1 did not affect the increase in LVEDP during reperfusion (Fig 7C).

#### Akt

To determine whether vancomycin-induced cardioprotection is mediated by Akt, isolated hearts were perfused with the Akt/PI_3_ kinase inhibitor wortmannin prior to ischemia and reperfusion. Wortmannin (100 nM) abolished the ability of vancomycin to reduce myocardial necrosis and to enhance ventricular function following ischemia/reperfusion. Wortmannin alone had no effect on cardioprotection (Fig 7A and 7B). Wortmannin did not affect the increase in LVEDP during reperfusion (Fig 7C).

#### Mitogen Activated Protein Kinases

We then determined whether vancomycin-induced cardioprotection is mediated by p42/44 and p38 MAPK. Hearts were perfused with the p42/44 MAPK inhibitor PD98059 prior to ischemia and reperfusion. PD98059 (10 μM) abrogated the ability of vancomycin to reduce myocardial necrosis and to enhance ventricular function following ischemia/reperfusion. PD98059 alone had no effect on cardioprotection (Fig 7A and 7B). PD98059 did not affect the increase in LVEDP during reperfusion (Fig 7C). Isolated hearts were also perfused with the p38 MAPK inhibitor SB 203580 prior to ischemia and reperfusion. SB 203580 (15 μM) abolished the ability of vancomycin to reduce myocardial necrosis and to enhance ventricular function following ischemia/reperfusion. SB203580 alone had no effect on cardioprotection (Fig 7A and 7B). SB203580 did not affect the increase in LVEDP during reperfusion (Fig 7C).

#### ATP-dependent potassium channels

To determine whether vancomycin-induced cardioprotection is mediated by K_ATP_ channels, hearts were perfused with a non-selective K_ATP_ channel blocker glibenclamide prior to ischemia and reperfusion. Glibenclamide (3 μM) abolished the ability of vancomycin to reduce myocardial necrosis and to enhance ventricular function following ischemia/reperfusion. Glibenclamide alone had no effect on cardioprotection (Fig 7A and 7B). Glibenclamide did not affect the increase in LVEDP during reperfusion (Fig 7C).

## Discussion

The results of the present study demonstrate that metabolites derived from the intestinal microbiota are linked to severity of myocardial infarction in the host. We used antibiotics as tools to decrease or increase the abundance of specific bacterial groups in the rat intestine. The changes in the composition of the intestinal microbiota caused by the antibiotics dramatically altered the metabolism of the host. Cluster analysis and pathway assignment showed that non-absorbed antibiotics reduced the levels of metabolites in multiple host metabolic pathways including: amino acids, γ-glutamyl amino acids, carbohydrates, energy production, cytidine containing pyrimidine, ascorbates and aldarate, and the xenobiotic benzoate and food components. By combing chromatography and mass spectrometry-based metabolite profiling platforms, we identified a panel of amino acid metabolites in the circulation associated with microbial fermentation of phenylalanine, tyrosine, and tryptophan in the intestines. Decreased levels of these aromatic amino acid metabolites caused by the antibiotics predicted decreased severity of injury from a future myocardial infarction. The cardioprotective effect of the antibiotics used is indirect as the antibiotics selected for use are not absorbed into the circulation; when added directly to the coronary perfusate, they have no effect on severity of myocardial infarction. Reconstitution of systemic aromatic amino acid metabolite levels, either through intravenous delivery or oral feeding, abolishes the cardioprotective phenotype. These results show that the reach of the metabolites produced by the intestinal microbiota can extend far beyond the local environment of the gut to affect the physiology of remote organs such as the heart. Our findings show metabolites derived from intestinal microbiota translocate to the circulation where they determine severity of injury from ischemia/reperfusion in heart. Thus the direction of flow of information is from the intestines to the heart. We have been unable to find studies that show signaling in the opposite direction, arising from ischemia/reperfusion injury in heart that alters microbial metabolites in the intestines or other remote microbial habitats.

Vancomycin treatment resulted in decreased abundance of *Clostridia*, one of the specific targets of this antibiotic, and increased abundance of *Bacilli* and *Proteobacteria*. In contrast, the antibiotic combination of streptomycin, neomycin, bacitracin and polymyxin B decreased *Bacilli* and had no effect on abundance of *Clostridia* and *Proteobacteria*. This finding suggests that it is not likely that altered abundance of individual groups of bacteria is responsible for cardioprotection in rats treated with the antibiotic combination. Similar reductions in aromatic amino acid metabolites observed with both the antibiotic combination and vancomycin treatment alone suggest that the intestinal microbiota responsible for cardioprotection are not restricted to a particular phylum. Based on our previous studies that showed an individual bacterium, *Lactobacillus plantarum 299v*, decreases severity of injury from an induced myocardial infarction [5], we propose that changes in the members of bacterial groups at the genus, species and strain levels are responsible for the cardioprotective phenotype. Further studies are needed to identify the specific intestinal microbiota that confer cardioprotection.

Higher levels of phenyllactate were seen in vancomycin-treated animals than in animals treated with the mixture of drugs. Phenyllactate is an antimicrobial compound with broad inhibitory activity against a range of food spoilage fungal and bacterial species. The lactic acid bacterium, *Lactobacillus plantarum*, produces phenyllactate at high levels, allowing it to survive competition with other microbes in the intestines. *Lactobacillus plantarum* also generates peptidoglycan precursors ending in D-lactate instead of D-alanine, making the bacterium intrinsically resistant to vancomycin. From this data it appears as though part of the changes in the intestinal microbiota of vancomycin-treated animals includes an over-proliferation of *Lactobacillus plantarum*. This supports our previous study showing this bacterium protects the heart against injury from ischemia-reperfusion [5]. We recently showed that *Lactobacillus plantarum* improves brachial artery flow-mediated dilation, a surrogate marker of cardiovascular risk, and decreases circulating levels of the inflammatory cytokines IL-8 and IL-12 in patients with stable coronary heart disease [24]. These findings indicate this bacterium may be developed to prevent and treat the progression of cardiovascular disease.

In our previous study, vancomycin decreased circulating leptin levels, resulted in smaller myocardial infarcts and improved recovery of post ischemic mechanical function as compared with untreated controls [5]. Thus decreased leptin levels appear to be unmasking a cardioprotective phenotype. Our current findings reveal vancomycin decreases, but does not eliminate, circulating levels of metabolites derived from aromatic amino acids, and decreases severity of injury from an induced myocardial infarction. We propose that decreasing the levels of aromatic amino acid metabolites in the circulation unmasks and activates cellular survival mechanisms. In support of this notion, inhibiting the protease-activated receptor 4 limits myocardial ischemia/reperfusion injury in rat hearts by unmasking adenosine signaling [25]. Similarly, decreasing glucose levels plays a critical role in unmasking propofol-induced cardioprotection [26]. Our results show vancomycin decreases phenylpropionate and phenylacetate. These two phenolic acid metabolites of phenylalanine increase mitochondrial dysfunction by interfering with NAD-dependent oxidation, increased production of reactive oxygen species and induced mitochondrial pore opening [27]. Thus untreated animals may exhibit an increased oxidative stress that increases the susceptibility of the myocardium to injury from infarction. Future studies are needed to determine if this mechanism is responsible for intestinal microbial metabolites mediating susceptibility to injury in the heart.

Microbial metabolites present in the circulation need to interact with the exterior of the cells of the heart in order to transduce their signals to the interior. Inhibition of JAK-2 and Src kinase prevents cardioprotection by vancomycin. JAK-2 transduces signaling events initiated by cytokines activating cell surface receptors of the heart to downstream molecules such as the signal transducer and activator of transcription (STAT) family members. Src kinase can be activated by, and serve as an effector of G-protein coupled receptors on the cell surface. Our findings suggest microbial metabolites are interacting with cell surface receptors to transduce their signals through Src kinase and the JAK/STAT pathway. Microbial metabolites may be interacting with other targets on the cell surface such as ion channels or ion exchangers to transduce their signal to the interior of the myocardium to confer a cardioprotective phenotype. Our findings suggest vancomycin is acting through known cell survival pathways in heart. Cardioprotection by vancomycin is mediated by activation of pro-survival kinases Akt, p42/44 MAPK and p38 MAPK, as well as activation of K_ATP_ channels, classical mediators and effectors of pharmacologic cardioprotection. These proteins are components of known intersecting and interdependent signaling pathways in the myocardium that determine severity of myocardial infarction. Based on our current findings of decreased circulating levels of metabolites derived from aromatic amino acids decreasing severity of injury from an induced myocardial infarction, we propose that decreased levels of these microbial metabolites are unmasking a cardioprotective phenotype. Additional studies are needed to identify the mechanism underlying the relationship between decreased levels of microbial metabolites and severity of injury from an induced myocardial infarction.

The proposed role of protein kinases and potassium channels in the signal transduction pathway by which microbial metabolites decrease severity of myocardial infarction has been based on experiments with kinase inhibitors, compounds that stop one kinase activating another, and potassium channel blockers applied at conventional inhibitory concentrations. This pharmacological approach is dependent on the relative specificity of the inhibitors and blockers used. For example, AG 490 primarily targets JAK2 activity, but it is also known to inhibit JAK1 and JAK-3 [28]. The pyrazolopyrimidine PP1 has been used to suggest physiological roles for the Src family protein kinases, although it does not discriminate between different members of this family [29, 30]. PP1 also blocks TGF-β-mediated cellular responses by directly inhibiting type I TGF-β receptors in a manner unrelated to Src signaling [31]. The fungal metabolite Wortmannin inhibits PI-3 kinases and also inhibits PI-4 kinase, MLCK and PLD [32]. Wortmannin can inhibit mTOR (mammalian target of rapamycin), another member of the PI-3 kinase superfamily [33]. PD98059 is a highly selective *in vitro* inhibitor of MEK1 activation and the MAP kinase cascade. PD98059 inhibits MEK1, an upstream kinase of p42/44 MAPK, and is an inefficient inhibitor of MEK2 [34, 35]. SB 203580 inhibits p38α, β and β2 (not γ and δ) MAP kinase by competing with the substrate ATP [36, 37]. While SB 203580 inhibits p38 activity, it does not significantly affect the activation of p38. SB 203580 does not inhibit PKA, PKC, MEKs, MEKKs, ERK or JNK MAP kinases [36, 38, 39]. The sulfonylurea glibenclamide is a non-selective ATP-dependent potassium channel inhibitor. Glibenclamide inhibits both Kir6.2/SUR1 and Kir6.2/SUR2A currents [40]. These pharmacological inhibitors may not be completely specific to target proteins, but when used cautiously can be used to assess the physiological roles of these enzymes and ion channels and identify signaling pathways for future investigation.

To maintain a symbiotic host-microbe relationship, the host must protect itself against microbial invasion. Disruption of this homeostatic coexistence is strongly associated with disease in the host. Several defense systems have evolved to protect the host against the microbial residents of the intestinal microbiota. Firstly, there is physical separation of microbiota from the host. The human and rat intestines are divided into discrete sections that spatially segregate host digestive activity in the stomach and small intestine, where microbial biomass is low, from high microbial biomass and enzymatic activity in the large intestine [41]. Secondly, several innate immune mechanisms work as a firewall to limit contact between the dense luminal microbial community and the intestinal epithelial cell surface. Goblet cells secrete mucin glycoproteins that assemble into a thick, stratified mucus layer. Bacteria are abundant in the outer mucus layer, whereas the inner layer is resistant to bacterial penetration. Epithelial cells (such as enterocytes, Paneth cells and goblet cells) secrete antimicrobial proteins that further help to eliminate bacteria that penetrate the mucus layer [42]. Should microbes breach the firewall, then phagocytic cells (neutrophils, macrophages), dendritic cells, natural killer cells and other innate lymphoid cells protect the host against injury.

The majority of current biomarkers for cardiovascular disease screening are based on pathways such as inflammation and cholesterol biosynthesis that are already known to be associated with cardiovascular disease. Consequently, available biomarkers frequently provide information that is associated with what is already known. Although associated biomarkers can underscore the importance of a biological pathway, they may not provide a substantial increase in diagnostic or predictive value. Thus there is a need for independent disease biomarkers that will provide additional clinical information derived from new biological axes. Current biomarkers are capable of detecting injury to heart during the evolution of a myocardial infarction. The present study describes a novel relationship between gut microbiota metabolites and the severity of future acute myocardial infarction. Microbial metabolites may be developed as biomarkers to predict future acute coronary events beyond established biomarkers.

### Conclusion

This study links gut microbiota metabolites to severity of myocardial infarction and may provide opportunities for novel diagnostic tests and interventions for prevention of cardiovascular disease. Understanding the relationships between the metabolites exported by the intestinal microbiota and the cardiovascular biology of the host has the promise to create another dimension of personalized medicine, where the flow of information between microbiome and host can lead to improved diagnostics and therapeutics. Microbial metabolites may illuminate novel pathways to disease and serve as biomarkers that can be assessed using novel diagnostic tests for susceptibility to and severity of myocardial infarction. Further novel therapeutic approaches using metabolite supplements can be devised for the treatment and prevention of myocardial infarction. Our studies support a new scientific paradigm that may lead to diagnostic and prognostic tests and thereby lead to highly innovative therapies in the treatment of myocardial infarction.

## Supporting Information

S1 Fig Examples of rat heart slices for measurement of infarct size. Viable tissue stains dark red when triphenyltetrazolium chloride reacts with intracellular dehydrogenases to form an insoluble red formazan dye. Infarcted tissue remains homogeneous white due to lack of staining of viable tissue. The border zones are clearly demarcated.
(PDF)

S1 File Mass spectrometry analysis. (PDF)

S1 Table Changes in metabolic pathways induced by antibiotic treatment. (PDF)

S2 Table Representative metabolites decreased by antibiotic treatments. (PDF)

S3 Table Primer sets specific for the 16S and 18S rRNA, reaction temperature and reference strains. (PDF)
