# Supplementary material for: Intestinal Microbial Metabolites Are Linked to Severity of Myocardial Infarction in Rats
Source: PLoS One. 2016 Aug 9;11(8):e0160840. doi: 10.1371/journal.pone.0160840 (PMC4978455; doi:10.1371/journal.pone.0160840)

Figure. Examples of rat heart slices for measurement of infarct size. Viable tissue stains dark red when triphenyltetrazolium chloride reacts with intracellular dehydrogenases to form an insoluble red formazan dye. Infarcted tissue remains homogeneous white due to lack of staining of viable tissue. The border zones are clearly demarcated.

Global ischemia - control

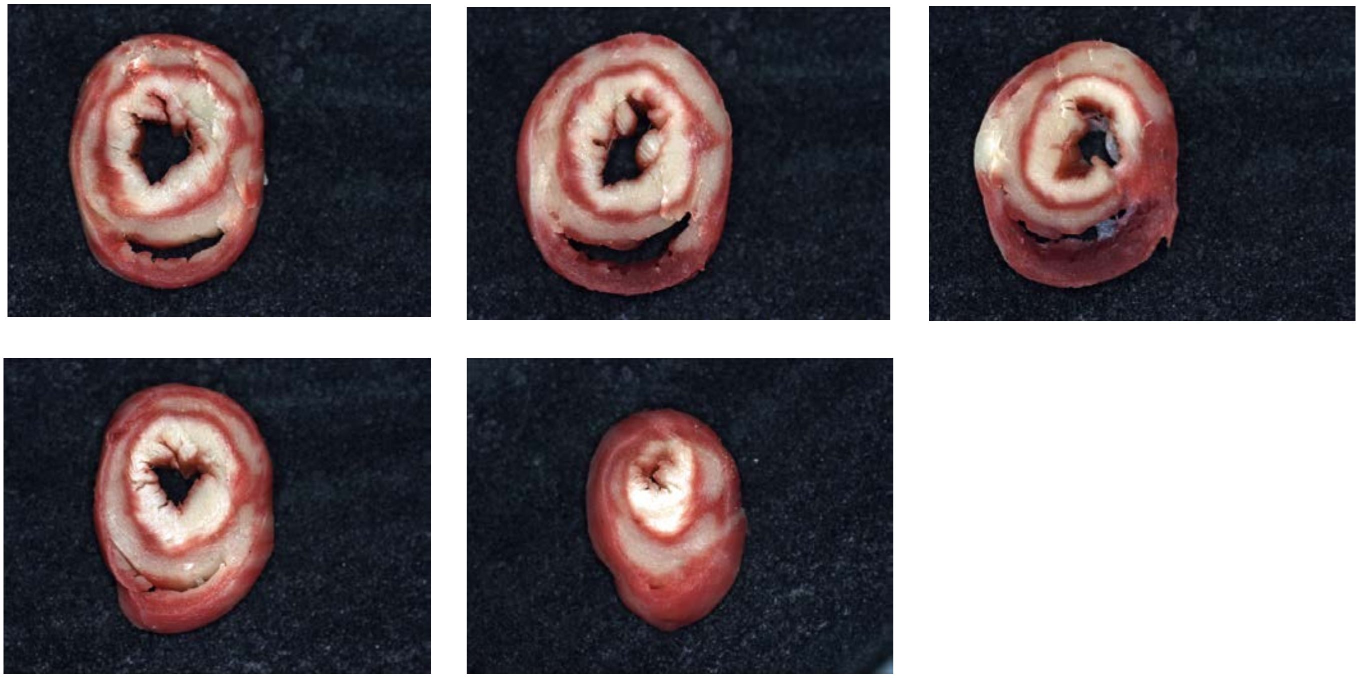

Global ischemia – vancomycin treated (60 mg/kg/day) for 7 days

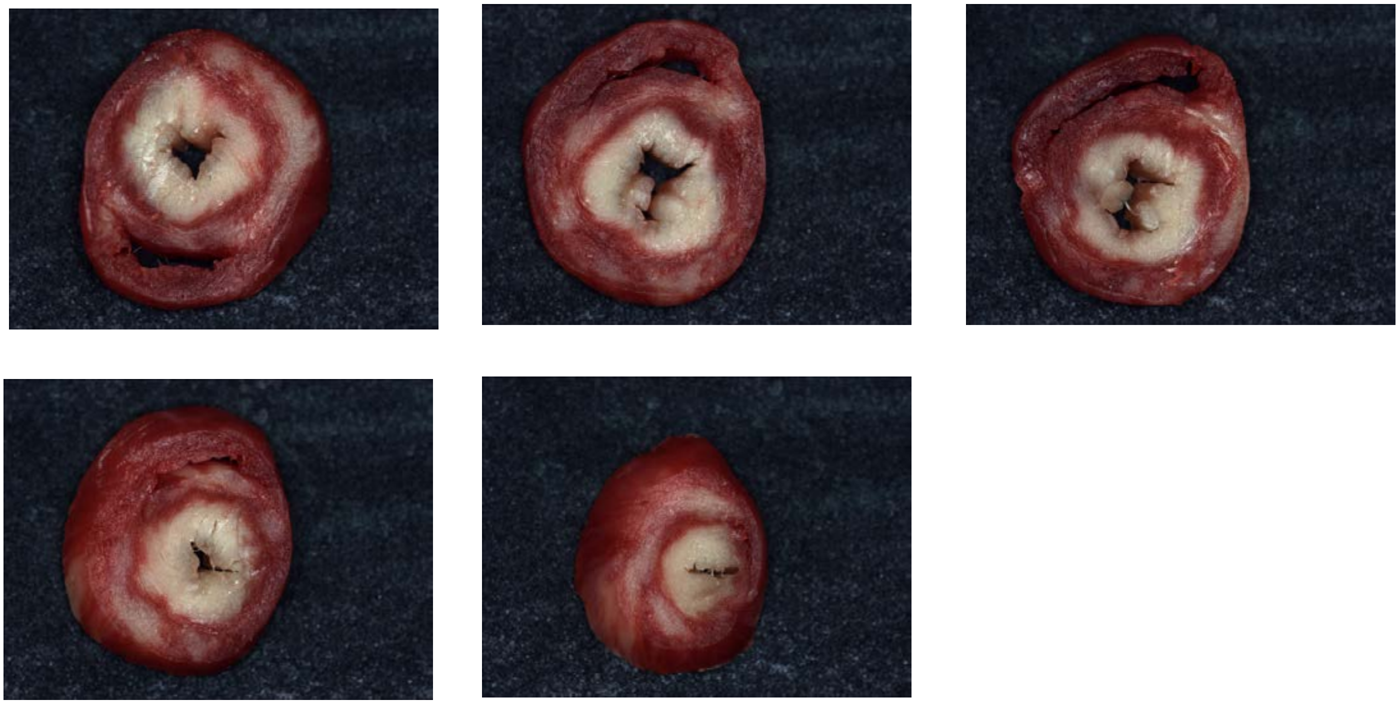

Regional ischemia - control

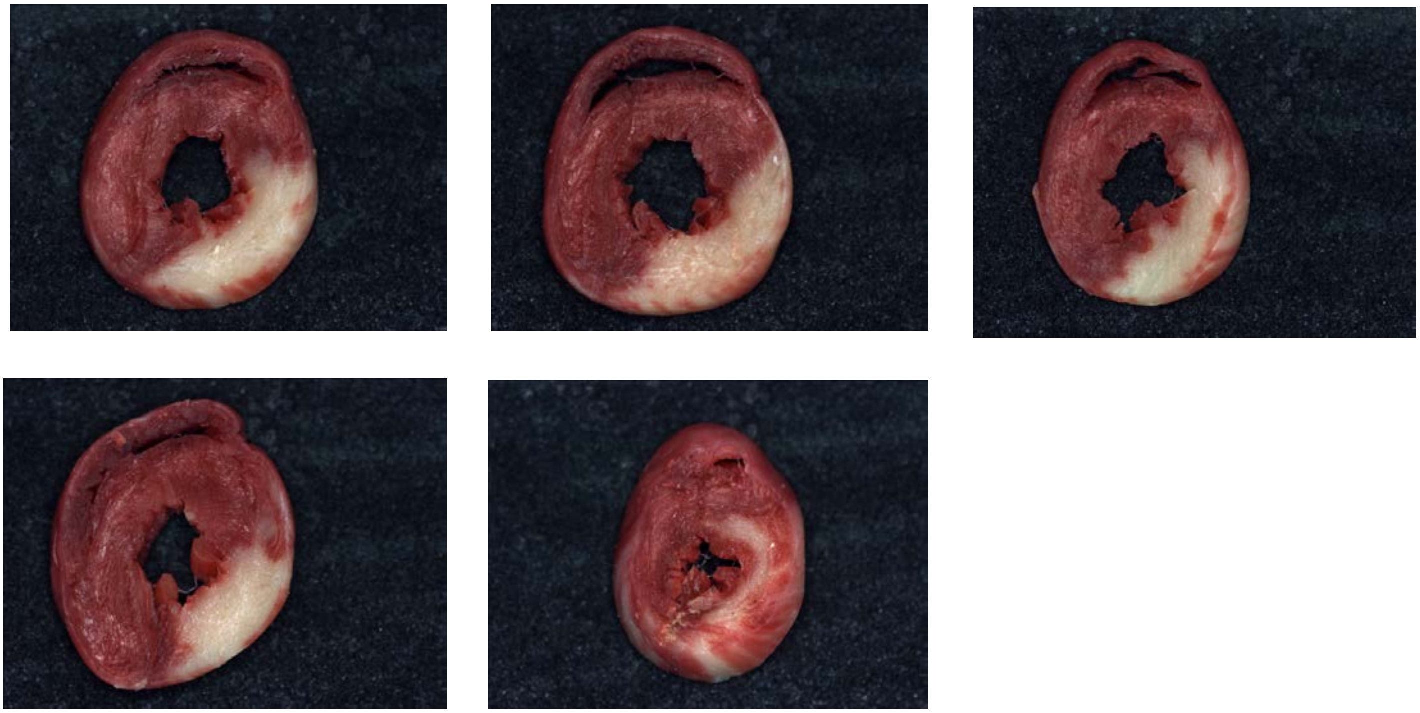

Regional ischemia – vancomycin treated (60 mg/kg/day) for 7 days

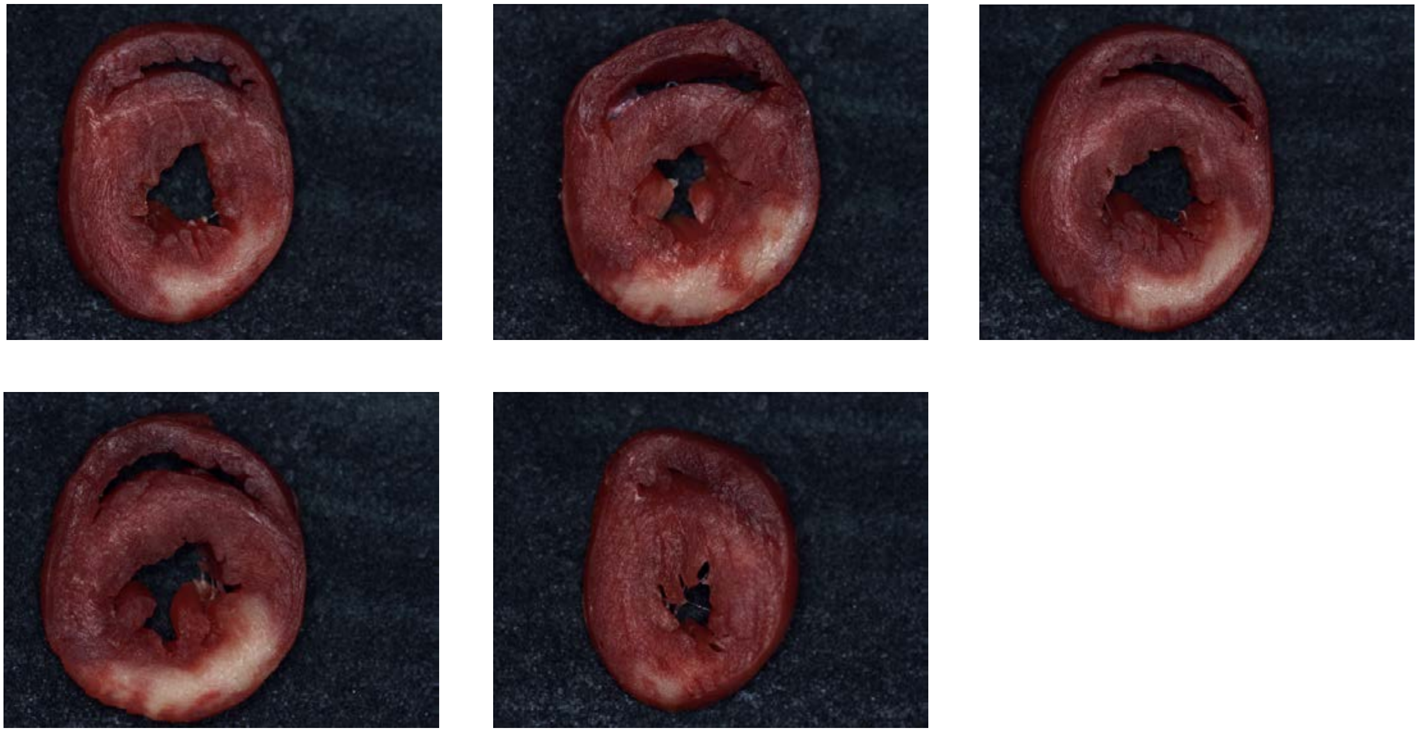

Supplement: S1 Fig — Viable tissue stains dark red when triphenyltetrazolium chloride reacts with intracellular dehydrogenases to form an insoluble red formazan dye. Infarcted tissue remains homogeneous white due to lack of staining of viable tissue. The border zones are clearly demarcated. (PDF) [file pone.0160840.s001.pdf]
