# Supplementary material for: Intestinal Microbial Metabolites Are Linked to Severity of Myocardial Infarction in Rats
Source: PLoS One. 2016 Aug 9;11(8):e0160840. doi: 10.1371/journal.pone.0160840 (PMC4978455; doi:10.1371/journal.pone.0160840)
Supplement: S1 File — (PDF) [file pone.0160840.s002.pdf]

Mass spectrometry analysis

Sample Accessioning

Each sample received was accessioned into the Metabolon Laboratory Information Management System (LIMS) system (Durham, NC) and was assigned by the LIMS a unique identifier, which was associated with the original source identifier only. This identifier was used to track all sample handling, tasks, results *etc.* The samples (and all derived aliquots) were bar-coded and tracked by the LIMS system. All portions of any sample were automatically assigned their own unique identifiers by the LIMS when a new task was created; the relationship of these samples was also tracked. All samples were maintained at -80 ºC until processed.

Sample Preparation

The sample preparation process was carried out using the automated MicroLab STAR® system from Hamilton Company. Recovery standards were added prior to the first step in the extraction process for quality control purposes. Sample preparation was conducted using a proprietary series of organic and aqueous extractions to remove the protein fraction while allowing maximum recovery of small molecules. The resulting extract was divided into two fractions; one for analysis by liquid chromatography (LC) and one for analysis by gas chromatography (GC). Samples were placed briefly on a TurboVap® (Zymark) to remove the organic solvent. Each sample was then frozen and dried under vacuum. Samples were then prepared for the appropriate instrument, either LC/MS or GC/MS.

Quality assurance/quality control (QA/QC)

For QA/QC purposes, a number of additional samples were included with each day's analysis. Furthermore, a selection of QC compounds was added to every sample, including those under test.

These compounds were chosen so as not to interfere with the measurement of the endogenous compounds. Tables A and B describe the QC samples and compounds. These QC samples were primarily used to evaluate the process control for each study as well as aiding in the data curation.

Table A: Description of Metabolon QC Samples

Type	Description	Purpose
MTRX	Large pool of human plasma maintained by Metabolon that has been characterized extensively.	Assure that all aspects of Metabolon process are operating within specifications.
CMTRX	Pool created by taking a small aliquot from every customer sample.	Assess the effect of a non-plasma matrix on the Metabolon process and distinguish biological variability from process variability.
PRCS	Aliquot of ultra-pure water	Process Blank used to assess the contribution to compound signals from the process.
SOLV	Aliquot of solvents used in extraction.	Solvent blank used to segregate contamination sources in the extraction.

Table B: Metabolon QC Standards

Type	Description	Purpose
DS	Derivatization Standard	Assess variability of derivatization for GC/MS samples.
IS	Internal Standard	Assess variability and performance of instrument.
RS	Recovery Standard	Assess variability and verify performance of extraction and instrumentation.

Liquid chromatography/Mass Spectrometry (LC/MS, LC/MS^2)

The LC/MS portion of the platform was based on a Waters ACQUITY UPLC and a Thermo-Finnigan LTQ mass spectrometer, which consisted of an electrospray ionization (ESI) source and linear ion-trap (LIT) mass analyzer. The sample extract was split into two aliquots, dried, then reconstituted in acidic or basic LC-compatible solvents, each of which contained 11 or more injection standards at fixed concentrations. One aliquot was analyzed using acidic positive ion optimized

conditions and the other using basic negative ion optimized conditions in two independent injections using separate dedicated columns. Extracts reconstituted in acidic conditions were gradient eluted using water and methanol, both containing 0.1% Formic acid, while the basic extracts, which also used water/methanol, contained 6.5mM Ammonium Bicarbonate. The MS analysis alternated between MS and data-dependent MS^2 scans using dynamic exclusion.

Gas chromatography/Mass Spectrometry (GC/MS)

The samples destined for GC/MS analysis were re-dried under vacuum desiccation for a minimum of 24 hours prior to being derivatized under dried nitrogen using bistrimethyl-silyl-triflouroacetamide (BSTFA). The GC column was 5% phenyl and the temperature ramp is from 40° to 300° C in a 16 minute period. Samples were analyzed on a Thermo-Finnigan Trace DSQ fast-scanning single-quadrupole mass spectrometer using electron impact ionization. The instrument was tuned and calibrated for mass resolution and mass accuracy on a daily basis. The information output from the raw data files was automatically extracted as discussed below.

Accurate Mass Determination and MS/MS fragmentation (LC/MS), (LC/MS/MS)

The LC/MS portion of the platform was based on a Waters ACQUITY UPLC and a Thermo-Finnigan LTQ-FT mass spectrometer, which had a linear ion-trap (LIT) front end and a Fourier transform ion cyclotron resonance (FT-ICR) mass spectrometer backend. For ions with counts greater than 2 million, an accurate mass measurement could be performed. Accurate mass measurements could be made on the parent ion as well as fragments. The typical mass error was less than 5 ppm. Ions with less than two million counts require a greater amount of effort to characterize. Fragmentation spectra (MS/MS) were typically generated in a data dependent manner, but if necessary, targeted MS/MS could be employed such as in the case of lower level signals.

Bioinformatics

The informatics system consisted of four major components, the LIMS, the data extraction and peak-identification software, data processing tools for QC and compound identification, and a collection of information interpretation and visualization tools for use by data analysts. The hardware and software foundations for these informatics components were the LAN backbone, and a database server running Oracle 10.2.0.1 Enterprise Edition.

LIMS

The purpose of the Metabolon LIMS system was to enable fully auditable laboratory automation through a secure, easy to use, and highly specialized system. The scope of the Metabolon LIMS system encompasses sample accessioning, sample preparation and instrumental analysis and reporting and advanced data analysis. All of the subsequent software systems were grounded in the LIMS data structures. It has been modified to leverage and interface with the in-house information extraction and data visualization systems, as well as third party instrumentation and data analysis software.

Data Extraction and Quality Assurance

The data extraction of the raw mass spec data files yielded information that could be loaded into a relational database and manipulated without resorting to BLOB manipulation. Once in the database, the information was examined and appropriate QC limits were imposed. Peaks were identified using Metabolon's proprietary peak integration software, and component parts were stored in a separate and specifically designed complex data structure.

Compound identification

Compounds were identified by comparison to library entries of purified standards or recurrent unknown entities. Identification of known chemical entities was based on comparison to metabolomic library entries of purified standards. As of this writing, more than 1000 commercially available purified

standard compounds had been acquired and registered into LIMS for distribution to both the LC and GC platforms for determination of their analytical characteristics. The combination of chromatographic properties and mass spectra gave an indication of a match to the specific compound or an isobaric entity. Additional entities could be identified by virtue of their recurrent nature (both chromatographic and mass spectral). These compounds have the potential to be identified by future acquisition of a matching purified standard or by classical structural analysis.
