# Supplementary material for: Intestinal Microbial Metabolites Are Linked to Severity of Myocardial Infarction in Rats
Source: PLoS One. 2016 Aug 9;11(8):e0160840. doi: 10.1371/journal.pone.0160840 (PMC4978455; doi:10.1371/journal.pone.0160840)
Supplement: S1 Table — (PDF) [file pone.0160840.s003.pdf]

Table 1: Changes in metabolic pathways induced by antibiotic treatment.

Pathway - Sub-pathway[1]	Metabolites[2] Total	Down	%[3]	Up	%[3]	Special[4]
Amino acid	85	56	66	7	8	
Glycine, serine and threonine metabolism	7	6	86			
Alanine and aspartate metabolism	7	5	71			
Phenylalanine & tyrosine metabolism	14	12	86			Phenyllactate (PLA)
Tryptophan metabolism	10	6	60	1	10	Kynurenate
Valine, leucine and isoleucine metabolism	16	10	63	2	13	
Cysteine, methionine, SAM, taurine metabolism	5	3	60			
Peptide	24	12	50	7	29	
Gamma-glutamyl	9	9	100			
Fibrinogen cleavage peptide	10			6	60	
Carbohydrate	10	6	60			
Fructose, mannose, galactose, starch, and sucrose metabolism	3	3	100			
Glycolysis, gluconeogenesis, pyruvate metabolism	5	3	60			Lactate
Energy	8	4	50			
Krebs cycle	6	4	67			
Lipid	115	26	23	39	34	
Essential fatty acid	7			6	86	
Medium chain fatty acid	8			1	13	
Long chain fatty acid	18			15	83	
Fatty acid, monohydroxy	4	1	25	3	75	
Fatty acid, dicarboxylate	4	3	75			
Carnitine metabolism	6	1	17	3	50	
Bile acid metabolism	11	9	82			
Lysolipid	32	5	16	1	3	
Nucleotide	15	9	60	1	7	
Pyrimidine metabolism, cytidine containing	5	3	60			
Cofactors and vitamins	10	7	70	2	20	
Ascorbate and aldarate metabolism	3	3	100			
Xenobiotics	17	9	53	2	12	
Benzoate metabolism	4	3	75			
Food component/Plant	6	4	67			

[1]Each metabolic pathway is divided into selected sub-pathways. Metabolite counts for the pathways are cumulative of all sub-pathways, see Supplement Table 2 for the complete list.

[2]Each of the 284 metabolites are assigned to each sub-pathway. Down indicates metabolites that are reduced by antibiotics and up indicates an increase.

[3]Percent of metabolites in each pathway / sub-pathway that changed at day 7 compared to day 0. Green indicates reduction while red indicates increase.

[4]These three metabolites exhibited opposite changes in direction in response to vancomycin and mixed antibiotic treatments, see text for discussion.
