# Supplementary material for: Intestinal Microbial Metabolites Are Linked to Severity of Myocardial Infarction in Rats
Source: PLoS One. 2016 Aug 9;11(8):e0160840. doi: 10.1371/journal.pone.0160840 (PMC4978455; doi:10.1371/journal.pone.0160840)
Supplement: S2 Table — (PDF) [file pone.0160840.s004.pdf]

Table 2: Representative metabolites decreased by antibiotic treatments

PATHWAY[1]	SUB-PATHWAY	BIOCHEMICAL NAME	Vanc 7d / Vanc 0d Ratio[2]	Vanc 7d / Vanc 0d *p*-Value[3]	Mix 7d / Mix 0d Ratio[2]	Mix 7d / Mix 0d *p*-Value[3]
Amino acid						
	Glycine, serine and threonine metabolism	glycine	0.76	0.013	0.85	0.126
		dimethylglycine	0.61	< 0.001	0.66	< 0.001
		serine	0.94	0.656	0.68	0.019
		threonine	0.56	< 0.001	0.52	< 0.001
		N-acetylthreonine	0.62	< 0.001	0.69	< 0.001
		betaine	0.67	< 0.001	0.70	< 0.001
	Alanine and aspartate metabolism	alanine	0.67	0.007	0.56	< 0.001
		N-acetyl-beta-alanine	0.88	0.001	0.83	< 0.001
		aspartate	0.63	0.005	1.00	0.912
		N-acetylaspartate (NAA)	0.72	0.086	0.55	0.007
		3-ureidopropionate	0.78	0.031	0.66	0.001
	Glutamate metabolism	glutamate	0.65	0.002	0.61	< 0.001
		glutamine	0.89	0.005	0.81	< 0.001
		pyroglutamine*	0.36	< 0.001	0.31	< 0.001
	Phenylalanine & tyrosine metabolism	phenyllactate (PLA)	1.69	< 0.001	0.14	< 0.001
		phenylalanine	0.87	0.013	0.86	0.008
		phenylacetate	0.78	0.046	0.84	0.137
		p-cresol sulfate	0.09	< 0.001	0.11	< 0.001
		tyrosine	0.95	0.193	0.88	0.003
		3-(4-hydroxyphenyl)lactate	0.95	0.307	0.81	< 0.001
		4-hydroxyphenylpyruvate	0.72	0.025	1.05	0.719
		N-acetyltyrosine	0.93	0.367	0.82	0.013
		phenylacetylglycine	0.19	< 0.001	0.93	0.942
		2-(4-hydroxyphenyl)propionate	0.22	< 0.001	0.17	< 0.001
		3-(3-hydroxyphenyl)propionate	0.54	0.045	0.35	0.024
		3-phenylpropionate (hydrocinnamate)	0.36	< 0.001	0.35	< 0.001
		phenol sulfate	0.04	< 0.001	0.03	< 0.001
	Tryptophan metabolism	kynurenate	0.81	0.005	1.21	0.016
		kynurenine	0.79	< 0.001	0.86	0.006
		indoleacetate	0.54	< 0.001	0.64	0.003
		serotonin (5HT)	3.11	< 0.001	0.85	0.657
		N-acetyltryptophan	0.74	0.017	0.75	0.014
		C-glycosyltryptophan*	0.87	0.004	0.89	0.009
		3-indoxyl sulfate	0.66	0.077	0.47	0.001
		indolepropionate	0.37	< 0.001	0.44	< 0.001
	Valine, leucine and isoleucine metabolism	3-methyl-2-oxobutyrate	0.82	0.040	0.98	0.815
		3-methyl-2-oxovalerate	0.71	0.010	0.80	0.253
		beta-hydroxyisovalerate	1.10	0.327	1.51	0.007
		isoleucine	0.81	0.003	0.91	0.101
		leucine	0.84	< 0.001	0.92	0.033
		N-acetylleucine	0.94	0.391	0.82	0.020
		valine	0.92	0.040	1.01	0.807
		2-hydroxyisobutyrate	0.72	< 0.001	0.63	< 0.001
		3-hydroxyisobutyrate	0.70	0.007	0.81	0.179
		alpha-hydroxyisovalerate	1.71	< 0.001	0.81	0.057
		isovalerylglycine	0.80	0.031	0.84	0.075
		2-methylbutyroylcarnitine	0.87	0.036	1.07	0.253
	Cysteine, methionine, SAM, taurine metabolism	methionine	0.84	< 0.001	0.81	< 0.001
		N-acetylmethionine	0.81	< 0.001	0.72	< 0.001
		2-hydroxybutyrate (AHB)	0.47	< 0.001	0.56	< 0.001
Peptide						
	gamma-glutamyl	gamma-glutamylvaline	0.78	< 0.001	0.89	0.069
		gamma-glutamylleucine	0.76	< 0.001	0.89	0.043
		gamma-glutamylisoleucine*	0.80	0.002	0.80	0.002
		gamma-glutamylmethionine	0.72	0.004	0.67	0.002
		gamma-glutamylglutamine	0.74	< 0.001	0.70	< 0.001
		gamma-glutamylphenylalanine	0.75	< 0.001	0.75	< 0.001
		gamma-glutamyltyrosine	0.78	< 0.001	0.75	< 0.001
		gamma-glutamylthreonine*	0.61	< 0.001	0.47	< 0.001
		gamma-glutamylalanine	0.74	< 0.001	0.72	< 0.001
Carbohydrate						
	Fructose, mannose, galactose, starch, and sucrose metabolism	fructose	0.59	< 0.001	0.61	< 0.001
		mannitol	0.92	0.843	0.06	< 0.001
		mannose	0.87	0.132	0.72	0.003
	Glycolysis, gluconeogenesis, pyruvate metabolism	1,5-anhydroglucitol (1,5-AG)	0.53	< 0.001	0.49	< 0.001
		glycerate	0.69	0.046	0.81	0.195
		glucose	0.71	0.003	0.67	< 0.001
		lactate	0.63	0.005	1.39	0.037
Energy						
	Krebs cycle	citrate	0.68	< 0.001	0.71	< 0.001
		cis-aconitate	0.69	< 0.001	0.64	< 0.001
		succinate	0.37	< 0.001	0.66	0.035
		malate	0.48	< 0.001	0.58	< 0.001
Nucleotide						
	Pyrimidine metabolism, cytidine containing	cytidine	0.88	0.054	0.81	0.003
		5-methylcytidine	0.91	0.018	0.87	< 0.001
		2'-deoxycytidine	0.94	0.010	0.89	< 0.001
		uracil	0.67	0.012	0.99	0.999
		uridine	0.81	< 0.001	0.83	< 0.001
		pseudouridine	0.98	0.370	0.93	0.013
Cofactors and vitamins						
	Ascorbate and aldarate metabolism	gulono-1,4-lactone	0.51	0.003	0.58	0.005
		ascorbate (Vitamin C)	0.55	0.184	0.38	0.030
		threonate	0.55	< 0.001	0.42	< 0.001
Xenobiotics						
	Benzoate metabolism	hippurate	0.06	< 0.001	0.06	< 0.001
		catechol sulfate	0.03	< 0.001	0.02	< 0.001
		4-ethylphenylsulfate	0.08	< 0.001	0.08	< 0.001
	Food component/Plant	indoleacrylate	0.43	< 0.001	0.48	< 0.001
		cinnamate	0.48	< 0.001	0.35	< 0.001
		stachydrine	0.59	< 0.001	0.62	< 0.001
		homostachydrine*	0.47	< 0.001	0.50	< 0.001
Lipid						
	Fatty acid, dicarboxylate	2-hydroxyglutarate	0.51	0.013	0.94	0.495
		tetradecanedioate	0.81	0.032	0.97	0.861
		hexadecanedioate	0.75	0.029	0.72	0.014
	Bile acid metabolism	cholate	0.01	< 0.001	0.00	< 0.001
		12-dehydrocholate	0.07	< 0.001	0.07	< 0.001
		glycocholate	0.03	< 0.001	0.06	< 0.001
		taurocholate	0.66	0.127	0.41	0.005
		3-dehydrocholate	0.30	0.017	0.47	0.108
		deoxycholate	0.14	< 0.001	0.21	0.003
		taurodeoxycholate	0.42	< 0.001	0.50	0.001
		beta-muricholate	0.01	< 0.001	0.04	< 0.001
		alpha-muricholate	0.13	0.024	0.17	0.040

[1] Comprehensive list of all pathways are shown in supplement table 1.

[2] Change reflected as ratio of 7 days (7d) relative to day 0 (0d, prior to antibiotic treatment). Vanc - vancomycin, Mix - mixed antibiotics.

[3] Significance indicated by repeated measure ANOVA test.
