# Supplementary material for: Intestinal Microbial Metabolites Are Linked to Severity of Myocardial Infarction in Rats
Source: PLoS One. 2016 Aug 9;11(8):e0160840. doi: 10.1371/journal.pone.0160840 (PMC4978455; doi:10.1371/journal.pone.0160840)
Supplement: S3 Table — (PDF) [file pone.0160840.s005.pdf]

Table 3. Primer sets specific for 16S and 18S rRNA, reaction temperature and reference strains

Kingdom	Phylum	Class	Taxonomy Order	Family	Genus	Species	Primer ID		Temp	Control sp.	Catalog	Fname	Forward	Rname	Reverse	Citation
Bacteria							Universal Bacteria		63	Ruminococcus productus	ATCC 27340D-5	UniF340	ACTCCTACGGGAGGCAGCAGT	UniR514	ATTACCGCGGCTGCTGGC	10
	Actinobacteria	Actinobacteria	Bifidobacteriales (20)				Bifidobacteriales		59	Bifidobacterium longum - Infantis	ATCC 15697D-5	BifiF143	CTCCTGGAAACGGGTGGT	UniR338	GCTGCCTCCCGTAGGAGT	10
	Deferribacteres															
	Fusobacteria (12)															
	Bacteroidetes						Bacteroidetes		61	Bacteroides fragilis	ATCC 25285D	BactF285	GGTTCTGAGAGGAGGTCCC	UniR338	GCTGCCTCCCGTAGGAGT	10
		Bacteroidia	Bacteroidales (136)	Rikenellaceae (15)												
				Bacteroidaceae (45)												
				Prevotellaceae (36)												
				Porphyromonadaceae (21)												
	Firmicutes															
		Bacilli (92)					Bacilli		56	Lactobacillus acidophilus	ATCC 4357D-5	LabF362	AGCAGTAGGGAATCTTCCA	LabR677	CACCGCTACACATGGAG	10
			Bacillales (18)	Bacillaceae (14)	Bacillus											
				Staphylococcaceae (4)	Staphylococcus			Staphylococcus	57.5	S. aureus	ATCC 700699D-5	g-Staph-F	TTTGGGCTACACACGTGCTACAAT	g-Staph-R	AACAACTTTATGGGATTTGCWTGA	18
			Lactobacillales (74)	Lactobacillaceae (36)	Lactobacillus	L. rhamnosus										
						L. plantarum		L plantarum	59	Lactobacillus plantarum	ATCC 8014D-5	Lpla-3	ATTCATAGTCTAGTTGGAGGT	Lpla-2	CCTGAACTGAGAGAATTTGA	21
				Enterococcaceae	Enterococcus			Enterococcus	53	E. faecalis	ATCC 700802D-5	g-Encoc-F	ATCAGAGGGGGATAACACTT	g-Encoc-R	ACTCTCATCCTTGTTCTTCTC	17
				Streptococcaceae (32)	Streptococcus			Streptococcus	55	S. thermophilus	ATCC BAA-250D	g-Str-F	AGCTTAGAAGCAGCTATTCATTC	g-Str-R	GGATACACCTTTCGGTCTCTC	17
		Clostridia					Clostridia		60	Ruminococcus productus	ATCC 27340D-5	UniF338	ACTCCTACGGGAGGCAGC	CcocR491	GCTTCTTAGTCAGGTACCGTCAT	10
			Clostridiales (669)	Clostridiaceae (212)	Clostridium	C. difficile										
						C. perfringens										
						C. leptum (IV)										
				Ruminococcaceae	Ruminococcus											
				Eubacteriaceae (276)	Eubacterium	E. rectale (XIVa)										
					Blautia	C. coccoides (XIVa)										
						R. productus										
		Erysipelotrichi														
	Tenericutes	Mollicutes (42)					Mollicutes		60	M. pneumoniae	ATCC 15531D	GPO-3	GGGAGCAAACAGGATTAGATACC	MGSO	TGCACCATCTGTCACTCTGTTAACCTC	22
			Mycoplasmatales	Mycoplasmataceae	Mycoplasma											
					Ureaplasma											
			Entomoplasmatales	Spiroplasmataceae	Spiroplasma											
			Acholeplasmatales	Acholeplasmataceae	Acholeplasma											
	Proteobacteria (124)						Proteobacteria		67	Escherichia coli	ATCC 10798D-5	Uni515F	GTGCCAGCMGCCGCGGTAA	Ent826R	GCCTCAAGGGCACAACCTCCAAG	10
		Betaproteobacteria (19)														
		Gammaproteobacteria (74)														
			Enterobacteriales (51)	Enterobacteriaceae	Escherichia	Escherichia coli										
						E. coli (eaeA)										
			Pseudomonadales (11)													
			Aeromonadales (4)													
Fungi	Ascomycota	Saccharomycetes	Saccharomycetales	Saccharomycetaceae												
					Candida		Candida		57	C. albicans	ATCC 14053D	CandidaF	TCGCATCGATGAAGAACGCAGC	CandidaR	TCTTTTCCTCCGCTTATTGATATGC	13
					Saccharomyces		Saccharomyces		54	S. cerevisiae	ATCC 9763D	Sacc-F	ATTGCTGGCCTTTTCATTG	Sacc-R	CGCCTAGACGCTCTCTTCTTAT	15
						S. boulardii										
						S. cerevisiae										
		Eurotiomycetes	Eurotiales	Trichocomaceae	Aspergillus		Aspergillus		55	A. flavus	ATCC 9643D-2	AsperF	CTGTTAGTGCGGGAGTTCAAATTC	AsperR	AACACCTGACCTTTCGCGTGTA	16
						A. fumigatus										
						A. flavus										
						A. niger										
						A. terreus										
					Penicillium											
						P. citrinum										
						P. sartoryi										
						P. westlingii										
	Microsporidia (protozoa)						Microsporidia		60	E. intestinalis	ATCC 50651	ProtoF(V1)	CACCAGGTTGATTCTGCCTGAC	ProtoR(PMP2)	CCTCTCCGGAACCAAACCCTG	19, 20
			Apansporoblastina	Enterocytozoonidae	Enterocytozoon	E. bieneusi										
				Unikaryonidae	Encephalitozoon	E. intestinalis										
Archea	Euryarchaeota (3)	Methanobacteria	Methanobacteriales	Methanobacteriaceae	Methanobrevibacter	M. smithii		M smithii	57	M. smithii	Dr. Jeff Gordon	MsmithF	CCGGGTATCTAATCCGGTTC	MsmithR	CTCCCAGGGTAGAGGTGAAA	14
